# Supplemental Report from the Committee of Practical Medicine

**Published:** 1867-08

**Authors:** L. T. Hewins

**Affiliations:** Loda, Ill.


CHICAGO MEDICAL EXAMINER.

N. S. DAVIS, M.D., Editor.

VOL. VIII. AUGUST, 1867. NO. 8.

### Original Contributions.

### SUPPLEMENTAL REPORT FROM THE COMMITTEE OF PRACTICAL MEDICINE.

By. L. T. HEWINS, Loda, Ill.

Read to the Illinois State Medical Society, June, 1867.

By special request of the chairman of your committee on practical medicine, it has become my duty to prepare a report especially for the eastern and southern portions of the State.

The constitutional provisions for our Society make it lawful that reference should be made, "as occasion requires, to medical topography," and, by implication, to the geography and geology of those portions of country that are within range of our investigations, as well as note "what changes have taken place in this State in the management of individual diseases; report the progress of epidemics and the character of prevailing diseases in special localities."

In order to present the subjects to be treated in this report with proper clearness, (particularly so when we come to treat of diseases in special localities,) it seems legitimate to refer briefly, if we may without being thought tedious, to the geography, geology, and topography of that part of our State to which this paper refers, comprising, as it does, an important part of that great hydrographical basin, known as the Mississippi Valley, extending from eastern to western mountain range.

The State of Illinois lies between 37° and 42° 30′ north latitude, and between 87° 30′ and 91° 40′ west longitude, embracing about 55,500 square miles, resembling, somewhat, an irregular triangle, the apex of which rests at the junction of the Ohio and Mississippi rivers, in latitude 37° north, and at an altitude of only about 300 feet above the Gulf of Mexico. Extending north, towards the base of the triangle, the face of the country is generally undulating; and, as it stretches out between its eastern and western boundaries, it becomes a vast prairie of table lands, reaching an altitude of more than 500 feet above the confluence of the Ohio and Mississippi rivers, beautifully interspersed with groves and belts of timber, especially along the streams that are tributaries to our natural boundaries. The general inclination of this vast plane is from north to south, as is indicated by the course of its rivers.

These table lands form, as it were, a dividing ridge through the middle and southern portions of the State, so that the waters shed off both towards the east and south-east, and west and south-west, emptying the accumulating waters in the State into the Mississippi from the western slope, and into the Ohio and Wabash from the eastern slope.

The upper waters of most of the streams which are northern and western tributaries of the Ohio and Wabash have their origin in ponds, or small lakes, and extensive marsh lands, and, not unfrequently, marshes are developed in the bottoms along the course of the streams, which, in many places, are wide, subject to spring and summer inundations, leaving them *bayous*, marshes, and ponds, abounding in drift and other foul deposits. The fertility of these bottoms and the luxuriant growth of vegetation are coextensive with the miasm which overshadows this vast belt of country, extending, as they frequently do, for miles in width from either shore of the stream. Eminently true is this of the Wabash bottoms. The water in these bayous and ponds dries up during hot weather in summer, and the drift and foul deposits from spring floods decay.

Surrounded, as the inhabitants of these table lands and bottoms are, with foul and mephitic air, their princely summer harvests are succeeded by autumnal fevers, both mild and malignant.

Among the more important streams meriting notice, in this section of country, are Big Bay Creek, Saline, Little Wabash, Embarrass, and Vermilion, with their tributaries, emptying their waters into the Wabash and Ohio.

This whole eastern and southern slope, to which reference is had, presents, in a geological point of view, so far as known, the sandstone formation, overlaid by coal and limestone treassures, which occasionally crop out in the breaks of the hills and near the bottoms along the water courses, as may be seen in Vermillion, Kankakee, LaSalle, and other counties. The upper beds of this whole district consist of clay, sand and gravel, with a covering of rich vegetable mould. The surface of the country, whether woodland or prairie, is generally undulating or level, seldom broken into hills and ravines.

The water which is used for domestic purposes by the people living on the eastern and southern slope of this table land, is what is called *hard;* a few inhabitants only, of the vast population which occupy this territory, use *cistern water.*

There are many mineral springs and wells in this district, some containing valuable medicinal properties, others quite insalubrious. Those of most interest, in a sanitary point of view, may be found in Iroquois County. During the last ten years, about 200 artesian wells have been obtained by boring, at an average depth of about 75 feet. From these wells, good, salubrious, chalybeate water is obtained in great abundance, both for man and beast. From the use of this artesian water, the health of the people has been improved at least one hundred per cent.

Your reporter has given particular attention to the geographical, geological, and topographical delineations of that part of State for which he is to write, so that a more comprehensive view may be obtained of the etiology of the diseases which prevail, and to aid in getting a clearer understanding of its medical history. Impressed, as he is, with the fact that there are diseases which are a common scourge of the human race, which seem independent of all known external influences, and present substantially the same symptoms, and are not materially influenced by *ease*, by *age*, or climate; there are others, influenced by known causes, such as inflammations from mechanical injuries, burns, fractures, etc.; and yet another class, resulting from a specific poison, such as *variola*, *rubeola*, etc. A knowledge of these diseases, their symptoms, lesions, and treatment, are, in a great measure, the common inheritance of the profession. On the other hand, there are diseases which seldom occur, except in climates or localities especially favorable for their production, or influenced by special states or conditions of society, among which may be mentioned bilious intermittent and remittent fever, typho-malarial fever, pneumonia, rheumatism, scurvy, etc. To the study of each class of these diseases the medical philosopher will give due consideration, as he must of necessity meet them in his professional career.

The diseases which are the common heritage of the race, prevail on the table land, and in the valleys of our State; besides these, we meet with those that are influenced by climate and locality. The more frequent in occurrence, are billious intermittent and remittent fevers. The milder forms prevail, mainly, on the table lands, and the more malignant are generally found in the low lands and in the bottoms.

In a practice of ten years, in my present locality, at an altitude of nearly 300 feet above Cairo, I do not remember to have seen a single case of *malignant* or *congestive chill*; and yet, during that time, I have treated many hundred cases of the milder forms of autumnal fever. But previous to 1856, while residing in the bottom lands of the Wabash and Ohio rivers, at an altitude of 300, and sometimes 400, feet lower than my present locality, I had frequent opportunities to study congestive chill by the bedside, in the fall and winter months; cases in which the system had received such a shock from the malarial poison that, at the very onset of the disease, all the vital forces seemed paralyzed, and the patient would die in a few hours, with or without treatment. As soon as the chill began, it would seem that the capillary system gave way, and the watery portions of the blood were forced into the alimentary canal, with enough of the coloring matter of the blood mingled therewith, so that the matter ejected from the stomach and bowels gave to the friends of the patient the idea of a fatal hemorrhage, which was indeed true. The best efforts of the medical man to stay the ravages of the malady were of no avail. When such facts are presented to us, we are forced to the conviction that the poison, whatever it may be, exists in its most virulent forms in places of low altitude, in the bottom lands, beside the bayous, and the marshes near our larger streams. As we recede from the low lands and reach higher altitude, the milder and more manageable forms of bilious fever take the place of the more malignant type; and as we ascend the plane, typho-malarial or genuine typhoid fever takes the place of autumnal fever. I do not mean to say that typhoid fever does not exist on the low lands, but that it is more prevalent on the table lands.

To get a truthful exposition of the diseases which have prevailed in the district under consideration during the past year, I addressed letters of inquiry to more than one hundred medical men in my district; from a very few only have I been honored with a reply. From those who have kindly contributed to the material continued in this report, I have the united testimony that the year just closed has been the most healthy of any for many years preceding it; more free from epidemics and other diseases, incident to this country.

Bilious intermittents and remittents have prevailed throughout the district for which I report, only to a very limited extent. Dr. J. P. TAGGART, of Cairo, writes, that in the country forty and fifty miles above Cairo, the people suffered from intermittent fever; the people in and about Cairo suffered comparatively little. Dr. E. W. MILLS, of Moultrie Co., writes, that malarial type of fever is the prevailing disease in his locality. Dr. PAYNE, of Clark Co., writes, that bilious fevers are common in the low lands, but in the up lands, typhoid is more frequent in autumn. Dr. P. H. BARTON, of Vermillion Co., writes, that malarial fevers prevailed to a limited extent in that locality. A fact, which the Dr. brought to my notice, worthy of consideration, is, that those who work in the coal-mines never have intermittent or remittent fever, proving, as Dr. B. suggested, that the real cause of bilious fever is of vegetable origin; but, as your reporter would say, proving that whatever the cause may be, the colliers may meet such gases or chemical agents in the mines as are capable of, and do completely neutralize the poison; for, I understand, the colliers are twelve hours of the twenty-four on land, and those, too, in which they would be most exposed to the disease—during the night and while they slept. While the miners were not at work, and above ground, they were subjected to the same influences as those were who constantly remained above ground, and were subject to autumnal fever. The gases of those mines will bear a more careful study in a sanitary point of view.

The treatment adopted for autumnal fevers, is pretty generally agreed upon and uniform:—Evacuants and antispasmodics, mild chloride of mercury, followed by cathartic, either oil or vegetable pills, and, during the intermission of fever, the free use of quinine and opium, as the patient will bear. My habit has been to give large doses of quinine and arrest the disease speedily, giving to an adult 4, 6, or 8 grains, repeated two, three, or four times if needed, in absence of fever. Thus, the disease is cut short, with less danger of a relapse.

In Iroquois County, there prevailed, during the month of August, a congestive form of remittent fever, which attacked both children and adults; with children, producing violent congestion of the brain; but with adults, congestion of liver, spleen, and stomach. The cases that were promptly cared for, recovered in six or eight days, under the following treatment: Emetics, antimony and ipecac.; evacuants, calomel, followed by castor-oil and turpentine; and when there was even partial remission in the fever, such doses of quinine as the age and condition of the patient would tolerate, with ice to the head, and frequent warm baths to the limbs and body. Those neglected, or poorly cared for, invariably died in from four to eight days from the onset of the attack.

A form of fever has existed in many parts of the district, somewhat resembling typhoid, but which lacks many of the characteristics of that disease, as described by those who see typhoid in hilly countries and high altitudes with a densely crowded population; but the disease resembles that called by Dr. G. B. WOOD, of Philadelphia, continued form of bilious fever; by Dr. A. CLARK, of New York, symptomatic fever; and, more recently, by the surgeons in the army, it has been well named typho-malarial. This disease has prevailed, alike, in the southern, middle, and northern parts of the State, particularly on the table lands. From August to January it is most prevalent, taxing the skill of the physician and strength of the patient in the most eminent degree.

*Symptoms.*—The patient complains for some days before being confined to bed, of loss of appetite, restlessness at night, pain in limbs and back, stiffness in the back of the neck, and general lassitude. After from two to four days, a chill comes on, of short duration, followed with fever and, not unfrequently, delirium, great heat and dryness of surface, dryness of tongue, and, generally, redness of the tongue, mouth and throat. The tongue becomes heavily coated after the first few days, first white, then yellow, and, as the disease advances, the coating is brown, and the tongue deeply fissured, so as sometimes to bleed. Sordes collect upon the gums and teeth. The same kind of coating which is seen upon the tongue, and in the mouth and throat, often extends to the larger air-passages in the lungs, and as the tongue and mouth are freed from this coating, it becomes loose in the lungs and is a source of much irritation in the air-passages. Resulting therefrom, we frequently have inflammation of the lungs, thus complicating the case and greatly compromising the patient's risk of recovery.

There are, usually, remissions in the fever, in about twelve, eighteen, twenty-four, or thirty-six hours, generally attended with some perspiration about the neck and shoulders, and at times all over the body; sometimes the whole surface will be moist or bathed with perspiration, even during the exacerbation of fever. Where perspiration is excessive, it is thought the patient is in more eminent danger.

The skin is hot (but not the burning heat of typhoid), moist, or dry, except when the patient has chill, which does not usually occur after the third day from the time the patient is confined to bed, but exacerbations and remissions of fever continue to a greater or less extent, throughout its entire course. If convalescence supervenes, the remissions are more distinct, until there is an entire intermission in the fever, the patient oscillating between fever and intermission, the fever period shortening daily, until it ceases altogether.

From the onset of the attack, the skin has a yellow or bronzed appearance; respiration hurried; the pulse increased in fulness and frequency, at first (with adults) during the fever not more than 90 beats in a minute, but as the disease advances, it reaches 100, 120, or even 140, sometimes open and full, and sometimes small, quick, and feeble; there is usually loss of appetite, at times amounting to loathing of food; sometimes nausea and vomiting, more frequently not—if there is vomiting, the ejections are sour or bitter, mostly yellow or green bile. There is usually great thirst, the patient calls for acidulated water or ice; the face is flushed; the eyes are dull and suffused; the patient, if not delirious, complains of headache, pain in the back and limbs, and is restless and wakeful. As the disease progresses, the pulse becomes more feeble and more frequent, seldom falling below 100, even during a remission; the tongue, dry, as before stated, generally cleans off after a week or ten days, the coating breaks up in large patches, and the tongue becomes smooth, red, and shines as if varnished, at times re-coated and deeply fissured, if the patient recover, to be again cleaned off and left red and appearing as if varnished, which condition often continues long after convalescence is established.

The bowels are usually constipated at first, but more easily acted upon by cathartics than with those who have autumnal fever. Sometimes there is diarrhœa from the onset, the alvine evacuations are commonly yellow, yellowish green, or black. In advanced stages of the disease, a troublesome diarrhœa is likely to set in, which exhausts the patient rapidly if not controlled. The bowels, at first, are usually about in a normal condition, except it be in the region of the liver and spleen there be a sense of fulness experienced by the patient, and a manifest prominence or hardness, especially in the right side, by passing the hand over the surface. As the disease progresses, the bowels become tympanitic and often painful, which causes the patient to lie on the back with the limbs flexed.

The urine is scanty, high-colored, *reddish*, or yellowish-brown, in advanced stages of the disease, giving a lateritious, mingled with a white deposit, with more or less mucus, but no albumen, that your reporter has ever been able to detect.

A fact worthy of notice is, that the epistaxis, the sudamina, and the rose-colored spots so frequently met with and described as belonging to typhoid fever, together with a tendency to excessive diarrhœa, are not so frequently met with in this form of disease. That these symptoms are occasionally present, collectively or singly, is not questioned; but that they are pathognomonic of the disease, and always present, is denied, for it has been the province of your reporter to attend patients, sick with the disease in question, for four, six, and eight weeks, without either nosebleed, diarrhœa, or the rose-colored spots. All ages and conditions in society are subjects of the disease, but those most obnoxious to it are from 15 to 30 years.

I have had but very few opportunities to be present at autopsical examinations of such as have died of this disease, so that my knowledge of the anatomical lesions caused by it is limited. The cases I have seen, invariably presented mucous inflammation of the stomach, duodenum, and small intestines; the mucous follicles in the duodenum are usually enlarged and sometimes ulcerated; Peyer's glands were frequently inflamed, but I have never found them ulcerated, as a result of this disease. The most marked appearance of the liver, revealed by the knife, is a mottled or slate color; somewhat over normal size; and harder than in health. The spleen was of reddish or black color. Of lesions in other parts of the system, I am not able to speak with clearness.

The general appearance of this disease in our State, during the fall and winter months, is my only apology for devoting so much of this report to its consideration, and the delineations above given are from my co-laborers, and from my own observations in the field where I reside.

*Treatment.*—There are few diseases flesh is heir to that demands the attention of the physician more than *typho-malarial fever*. It requires less medicine than other diseases, but unremitting vigilance on the part of the physician. Timely directed, gentle cathartics, diurectics, diaphoretics and tonics are the main agents employed. The poisoned condition of the blood, the result of a failure of one or more of the excretory organs to remove from the system the effete matter which is constantly accumulating from the changes in the living tissues. It may be the skin, the liver, or kidneys, or all of these combined, and thus retaining within the blood, excesses in carbonic acid, urea, coloring matter of the bile, and other poisoning material. Such a condition requires the use of those agents that will neutralize the poison, and eliminate it from the system. When the patient is first seen, if the bowels have not been moved for a day or two previous to the physician's visit, calomel, followed with castor oil and turpentine, are advised, so as to cause two or three evacuations, repeating the oil and turpentine, or tartrate of potash and soda, in drachm doses, until the bowels move, this only during the second or third day. The diurectic or diaphorectic with which I have been best pleased, is a solution of the citrate of potash made from formula found in U. S. D., given in tablespoonful doses, to an adult, every two or three hours, with warm bath during the continuance of the fever, with ice held in the mouth and swallowed, if the patient desire, and acidulated and mucilaginous drinks; if the patient's mind is clear, and yet restless, pulvis doveri, v. grs., at bedtime, repeated, if need be, to secure rest at night. During the remission of the fever, quinine is administered in two or three grain doses every two or three hours. While the fever lasts the alkali and warm bath are freely used. If, during the progress of the disease, the bowels become tympanitic, turpentine emulsion is freely used, one or two drachms during twenty-four hours. Alcoholic stimulants are used in the latter part of the disease, if the patient seems feeble and little or no inclination to take food, and, if needful, the quinine and opiates increased. If the lungs become involved, as they frequently do, the best agents in my hands to meet this complication have been the protracted use of the turpentine emulsion and solution of the carbonate of ammonia, from five to eight grain doses, repeated every four hours. Such is an outline of the treatment adopted by my colleagues and myself, with such variations as individual cases have required, and with this course persistently followed we have had the satisfaction of knowing that a very large per cent. of our patients recovered.

*Cerebro-Spinal Meningitis* has prevailed in different parts of the State. Wherever it has prevailed its ravages have been fearful. Dr. PAYNE, of Clark County, stated to me that in an epidemic of this disease which prevailed last spring, within his field of labor, at least three-fourths of the cases proved fatal. It has also prevailed in the north part of Kankakee, and the southern part of Will Counties, with great severity. Many cases have been met with in Ford and Iroquois Counties during the months of February and March last. Most of the cases that occurred in Ford and Iroquois Counties were several miles in the country, and when medical aid was obtained, the patients were most of them in *articulo mortis*, beyond the reach of medicine. During the months of August and September of 1865, I had an opportunity of studying this disease at the bedside. My patients were all children, from two to ten years of age. About fifty per cent. of the cases died, and but one or two of the cases that I saw entirely recovered, free from complications. Some got up with partial loss of the use of one or more limbs; others with distorted features, or the loss of some of the senses, either of sight, hearing, or taste.

The causes, symptoms, pathology, and treatment of this formidable malady have been so fully discussed before this Society on former occasions in the invaluable monographs now on record, written by the Chairman of your present Committee on Practical Medicine, by our present permanent Secretary, by Prof. JEWELL, of Chicago, and other gentlemen of eminent professional attainments in the State, that I feel relieved from this duty, except it may be competent to note a few points which will bear frequent repetition from their important relations to this subject. A noticable fact in all the cases that I have treated, when the patient was not in a perfectly comatose condition, was generally found on the back, resting most of the weight of the body on the occiput and lower extremities, the pupils of the eyes largely dilated, eyelids closed and limbs extended, and whenever approached with an attempt to move any part of the body, or even removing the bed clothes, the sufferer complained as if in great agony. There is always retention or suppression of urine, and a suspension of the normal function of the skin in the cases I have seen, and it has been my custom to resort at once to the hot bath, with mustard, if the surface is cool, and when the patient is removed from the bath, should be wrapped in flannel blankets, and the internal use of large doses of an approved preparation of tincture of cantharides, of from five to fifty drops, according to age, with chlorate of potash, in from two to ten grain doses, repeated every two hours, until a sensible impression is made on the kidneys. If strangury is produced, as cantharides may do, and it is hailed as a favorable rather than an unfavorable omen. In all the cases I have treated, if the skin and kidneys could be brought actively under the influence of remedial agents, the patient recovered. To get a more minute and complete history of this disease my hearers are referred to the papers before mentioned.

*Dysentery*, in an epidemic form, so far as I know, has not prevailed in the eastern and southern parts of the State, except in Edgar County, and perhaps some parts of Coles. I learn of the epidemic in that locality, from Drs. MASSIE and GEORGE RINGLAND. To the latter gentleman am I indebted for an extended report respecting the epidemic, whose language I feel at liberty to transcribe for the information of the Society.

Dr. RINGLAND reports that: "The first case came under treatment 28th June, and the last cases treated were in September. The principal part of them occurred during the months of July and August. About 100 cases were under treatment in a town of 600 inhabitants. Of the whole number sick 13 died. The majority of the cases were children, and the mortality greatest among them. Of the deaths, three were adults and ten children. The disease was principally confined to town, but few cases in the country. The disease presented the symptoms usually met with in dysentery. There was fever, tormina-tenesmus; small mucus and bloody discharges. The cause of the disease was local. There were several hundred bushels of corn, unsound when first put in open pens, and by exposure to the hot weather and the rains in the months of May and June, it soon became putrid, and could be scented at great distance from the crib, and from the atmosphere thus poisoned the epidemic doubtless had its origin. The treatment was in no way unusual. The remedial agents used were opium, ipecac, calomel, castor oil, turpentine, tonics, etc."

*Cholera* has prevailed more or less in many parts of the State, and in some portions extensively. Dr. TAGGART, of Cairo, has kindly furnished me the following history of the epidemic, as it prevailed in that city last summer. He reported "between 350 and 400 cases of pure Asiatic cholera as having occurred in Cairo, and out of that number not over ten per cent. of the cases proved fatal. It was surprising to find with what rapidity the disease appeared to leave, for its course was only about four or five weeks duration. The first symptoms were usually slight nausia, followed with transient pains or rumbling in the bowels, diarrhœa, slight and painless at first. Such I have invariably found to be but the skirmishing part of an advancing column, and if I would stay its ravages must not hesitate to commence at once with the following

"℞.—Tinct. opii, spirits camphor,------------āā ℥j.
Tinct. rhei,-------------------------------℥ij.
Chloroform,------------------------------℥ss.

Mix.

"Give teaspoonful every one-half hour until the diarrhœa is arrested, which will be on taking the third or fourth dose, when the case is taken in season. In more advanced cases, where the diarrhœa was more troublesome, injections of starch and laudanum were used. With cases of greater violence, to the foregoing mixtures there is added, in small doses, calomel, opium, and plumbi acetas, with free use of mustard cataplasms to the stomach, friction to the parts affected with cramps, requiring the patient at all times to lie still, on the back. Chloroform, in any stage of the disease, when freely used, by inhalation, will do much to relieve the suffering caused by cramping in the extremities."

The disease also prevailed in Edgar, Coles, Champaign, Iroquois, Cook, and probably other counties in the State. The causes, symptoms, pathology, and treatment of which will be abundantly and ably discussed by my colleague, the Chairman of your Committee, whose residence in the city of Chicago brought him in immediate relation with the disease, and afforded him an abundant opportunity for such investigation, to whose paper I feel at liberty to refer for extended views on this subject.

*Variola* has been much less prevalent the past than a few years just preceding, as the more fruitful sources for its spread have been sensibly diminished since the close of the war, and as there has doubtless been greater vigilance exercised to control it by vaccination and revacination, thus stopping, abruptly, its progross wherever it has appeared. The few cases I have seen were not permitted to spread, for all persons were at once vaccinated who were likely to be exposed in any way, confining the disease to its first subjects.

*Diptheria* has prevailed in some parts of the State in a troublesome form, but I think not as a serious epidemic. Many cases have occurred in the middle portion of the State, which, as I learn, have been successfully treated with chlorate of potash, tonics, and stimulants, internally; salt bath, externally, and the permanganate of potassa, used as a gargle. I think too much importance cannot be attached to the free use of the chlorates in the treatment of this formidable disease, as well as in the treatment of scarlet fever. During an epidemic of these diseases, which prevailed together in the years 1864

and 1865, in Iroquois County, about two hundred and fifty cases of both diseases came under my care, at which time I had repeated opportunities to test the virtues of the chlorates, and equally frequent opportunities to witness their good effects upon my patients.

*Rubeola* has not, to my knowledge, prevailed in the State as an epidemic, except in Champaign Connty, where, I learn from good authority, it prevailed during the three winter months, with great severity. It is reported that about one thousand persons fell victims to this malady. Many of the cases were of a malignant type; only about one-twentieth were adults. In a number of cases persons had the disease who had been its subjects before. Most of the fatal cases were the result of the complications. As there is always, with measles, inflammation of the skin and the mucous surfaces, so when it prevails in cold or damp weather, we are very likely to have complications. The most frequent are inflammation of the throat and lungs. Thus has it been with the epidemic in question. The fatality was the result of bronchitis or pneumonia. There seemed to be a special tendency to pneumonia, which was attributed to the extreme cold weather that we had during the prevalence of the disease; for when we had warm weather in the month of February there was a marked diminution in the pulmonic complications, and the disease assumed a much milder and more manageable form. The causes, symptoms, pathology, and treatment of this disease have been so thoroughly discussed in our textbooks, and are generally so well understood by the medical men that it seems useless to consume time in discussing these points here. Permit me to say, however, that in addition to the ordinary treatment adopted for measles, in *this epidemic* the sulphites (especially soda) were used freely and with marked success. Such is the condition of the skin and mucous membrane in this form of disease, that these surfaces fail to perform their natural functions, and, consequently, a large amount of effete matter, which in health is eliminated through the pores of the skin, from the lungs and kidneys; is retained in the blood at great risk to the patient; and, from the short experience had from the use of the sulphites, there are in use no agents so neutralizing to the accumulating poisons, and in preventing their destructive influences on the normal constituents of the blood. Their use, therefore, in the treatment of rubeola and other exanthematous diseases, is worthy of the careful consideration of the profession.

The sulphites of soda and lime, the permanganate of potash, the bromides of potash and ammonium, have all been recently brought into use as medical agents. Bromine and its compounds have been especially urged upon the notice of the profession as valuable disinfectants, antiseptics, and possessing in a wonderful degree a controlling influence over nervous affections.

Two *very valuable papers*, bearing upon the efficiency of the bromides in treating nervous affections, were read to the Society at our last Annual Meeting, both from distinguished members of our Society, each of which are worthy of careful study. During the last two years I have used bromide of ammonium with good results, in treating whooping cough, hysteria, epilepsy, and in protracted cases of pneumonia, where the lungs are left in an irritable condition. I have also found it serviceable in controlling nervous restlessness at night. Taking due care that *too much* is not expected from these agents, we may, I think, hope to use them with decided benefit, especially in treating nervous affections.

